# The impact of the combat method on radiomics feature compensation and analysis of scanners from different manufacturers

**DOI:** 10.1186/s12880-024-01306-4

**Published:** 2024-06-06

**Authors:** Xiaolei Zhang, M. Iqbal bin Saripan, Yanjun Wu, Zhongxiao Wang, Dong Wen, Zhendong Cao, Bingzhen Wang, Shiqi Xu, Yanli Liu, Mohammad Hamiruce Marhaban, Xianling Dong

**Affiliations:** 1 Faculty of Engineering, Universiti Putra Malaysia, Serdang, Malaysia; 2 Hebei International Research Center of Medical Engineering, Chengde Medical University, Chengde City, Hebei Province China; 3 Hebei Provincial Key Laboratory of Nerve Injury and Repair, Chengde Medical University, Chengde City, Hebei Province China; 4 Department of Radiology, the Affiliated Hospital of Chengde Medical University, Chengde, Hebei China; 5 Institute of Artificial Intelligence, University of Science and Technology Beijing, Beijing, China; 6 Department of Biomedical Engineering, Chengde Medical University, Chengde City, Hebei Province China

**Keywords:** Combat, Radiomics, Machine learning, Phantom, CT images

## Abstract

**Background:**

This study investigated whether the Combat compensation method can remove the variability of radiomic features extracted from different scanners, while also examining its impact on the subsequent predictive performance of machine learning models.

**Materials and methods:**

135 CT images of Credence Cartridge Radiomic phantoms were collected and screened from three scanners manufactured by Siemens, Philips, and GE. 100 radiomic features were extracted and 20 radiomic features were screened according to the Lasso regression method. The radiomic features extracted from the rubber and resin-filled regions in the cartridges were labeled into different categories for evaluating the performance of the machine learning model. Radiomics features were divided into three groups based on the different scanner manufacturers. The radiomic features were randomly divided into training and test sets with a ratio of 8:2. Five machine learning models (lasso, logistic regression, random forest, support vector machine, neural network) were employed to evaluate the impact of Combat on radiomic features. The variability among radiomic features were assessed using analysis of variance (ANOVA) and principal component analysis (PCA). Accuracy, precision, recall, and area under the receiver curve (AUC) were used as evaluation metrics for model classification.

**Results:**

The principal component and ANOVA analysis results show that the variability of different scanner manufacturers in radiomic features was removed (P˃0.05). After harmonization with the Combat algorithm, the distributions of radiomic features were aligned in terms of location and scale. The performance of machine learning models for classification improved, with the Random Forest model showing the most significant enhancement. The AUC value increased from 0.88 to 0.92.

**Conclusions:**

The Combat algorithm has reduced variability in radiomic features from different scanners. In the phantom CT dataset, it appears that the machine learning model’s classification performance may have improved after Combat harmonization. However, further investigation and validation are required to fully comprehend Combat’s impact on radiomic features in medical imaging.

**Supplementary Information:**

The online version contains supplementary material available at 10.1186/s12880-024-01306-4.

## Introduction

The United Nations Health Organization stated that out of the 19.2 million people with cancer in 2020, 9.9 million died. Radiomics has become a promising area of research for diagnosing, staging, and predicting tumors [1]. The radiomics workflow includes collecting images, preprocessing images, identifying regions of interest, extracting features, and building models [2]. In general, large-scale multicenter research must collect medical images from different sites and equipment. Many studies have demonstrated that radiomics features are sensitive and variable to scanners, scan parameters, and reconstruction algorithms [3–7]. This sensitivity and variability pose a significant challenge for the clinical application of radiomics [8, 9]. Furthermore, the variability in radiomics affects its subsequent statistical analysis and machine learning models. Therefore, more accurate radiomic features are extracted when noise is removed. Johnson and his colleagues proposed an empirical Bayesian function for nonparametric estimation (Combat) capable of adjusting batch effects in genetic data while preserving their biological properties [10]. In gene expression measurement experiments, different batches of experiments exhibit different environments and operating equipment, which create a ‘batch effect’ that invalidates the data. Radiomic features are also sensitive to conditions such as different acquisition equipment scan parameters.

Therefore, the Combat method can theoretically be applied to the removal of multi-center noise in radiomic features, which is the compensation of radiomic features. On the other hand, Fanny Orlhac and her colleagues demonstrated that the Combat algorithm successfully adjusted radiomics feature distributions computed from different CT imaging protocols and facilitated multicenter radiomics studies [11]. However, this study did not investigate whether the Combat algorithm affects the performance of subsequent statistical analysis of the radiomic machine learning model. In addition, Fortin, Jean-Philippe and his colleagues stated that applying the Combat algorithm to compensate for multi-site effects of voxels in diffusion tensor imaging eliminates site-to-site variability while preserving biological variability such as age [12]. They also applied Combat to measure cortical layer thickness based on MRI data from different sites. The authors argued that Combat reduced the variability among different scanners and improve its performance in subsequent statistical analysis [13]. On the other hand, Da-anol and his colleagues assessed the ability of Combat and modified B-Combat and M-Combat to compensate for radiomics at different centers. They demonstrated that Combat and the modified Combat methods remove differences through performance metrics of machine learning pipelines [14].

The datasets consist of radiological images from various scanner manufacturers, which can adversely affect the performance of radiomic machine learning models. Multicenter effect compensation studies are needed for CT images of different scanner manufacturers and different scan parameters. This study used an open-source dataset, which is a phantom data of different scan and reconstruction parameters on different models of CT [15]. The purpose of establishing this dataset was specifically to investigate the variability of radiomic features caused by different scanners and parameters.

(https://wiki.cancerimagingarchive.net/pages/viewpage.action?pageId=39879218).

Since applying the Combat method to multicenter compensation of radiomic features is a new field, many studies have focused on its compensatory effect on the radiomic characteristics. This study hopes to provide a reference for the subsequent modeling and analysis of the Combat method to compensate for the radiomics features of the multicenter effect. Cartridge regions of two different materials were marked as ROI, and radiomic features were extracted from the regions of interest. The impact of the Combat method on the distribution of radiomic features of scanners from different manufacturers was investigated. In addition, the study validated whether the Combat algorithm could improve the performance of subsequent modeling analysis of radiomic features. We hope that this study can provide some valuable insights for future research or applications of the Combat algorithm in radiomic machine learning classification.

## Materials and methods

### Dataset and preprocessing

The Credence Cartridge Radiomic (CCR) phantom dataset was collected from the public dataset. This dataset can be used to investigate the effects of scanners of different manufacturers on radiomic features. Table 1 shows that the Credence Cartridge had ten different material compositions representing different textures. Radiomic features extracted from different cartridges, such as rubber and resin, were defined as distinct categories. The machine learning model was constructed to differentiate between these two different categories of features.

**Table 1:** Credence cartridge phantom CT scan description

Cartridge number	Description
Cartridge 1	ABS plastic 20% honeycomb filling
Cartridge 2	ABS plastic 30% honeycomb filling
Cartridge 3	ABS plastic 40% honeycomb filling
Cartridge 4	ABS plastic 50% honeycomb filling
Cartridge 5	Sycamore wood
Cartridge 6	Rubber particles
Cartridge 7	Dense cork
Cartridge 8	Solid Acrylic
Cartridge 9	Natural cork
Cartridge 10	Plaster resin

This dataset used eight scanners from three manufacturers, Siemens, Philips, and GE Healthcare, to assess differences in radiomic features between manufacturers and scanners. In total, 251 CT cohorts were acquired using different reconstruction parameters, voltages, currents, slice thicknesses, and reconstruction kernels. Out of the 251 CT cohorts, 41 phantom cohorts with different pitches, 20 phantom cohorts with different currents, and 55 phantom cohorts with different reconstruction kernels were excluded.

As shown in Table 2, CT phantom images from each manufacturer have the same reconstruction kernel, scan current and voltage, scan type, reconstruction FOV. In this study, 135 CT phantom cohorts with 53 S, 42 Philips, and 40 GE were screened to investigate differences in radiomic features between manufacturers, and the study found differences in slice thickness. These studies have demonstrated that radiomic features are sensitive to many factors, including reconstruction kernel, reconstruction FOV, slice thickness, and pixel size. This study integrated other factors influencing radiomics characteristics into the manufacturer’s scanner grouping. Since it is impossible to control all the factors influencing radiomics characteristics, this study tried to attribute all the factors to noise from scanners with different manufacturers. The principal component analysis was used to justify the grouping by scanners from different manufacturers.

**Table 2:** CCR Phantom scans of different manufacturers (total = 135)

CT scanner	Reconstruction Kernel.	mAs	kVp	Scan type	Detector configuration (mm)	Varying reconstruction FOV (mm)
GE Discovery STE	Standard	250	120	Helical	Det. Coverage = 20	200, 250, 300, 350, 400, 450, 500
GE Lightspeed 32	Standard	250	120	Helical	Det. Coverage = 20	200, 250, 300, 350, 400, 450, 500
Philips Brilliance 64	Standard (B)	250	120	Helical	64 × 0.625	200, 250, 300, 350, 400, 450, 500
Philips Big Bore 16	Standard (B)	250	120	Helical	64 × 0.625	200, 250, 300, 350, 400, 450, 500
Siemens Sensation 64	B31f	250	120	Helical	64 × 0.6	200, 250, 300, 350, 400, 450, 500
Siemens Sensation 40	B31f	250	120	Helical	40 × 0.6	200, 250, 300, 400, 500
Siemens Sensation 16	B31f	250	120	Helical	16 × 0.6	200, 250, 300, 400, 500

### Image segmentation

**Fig. 1:**
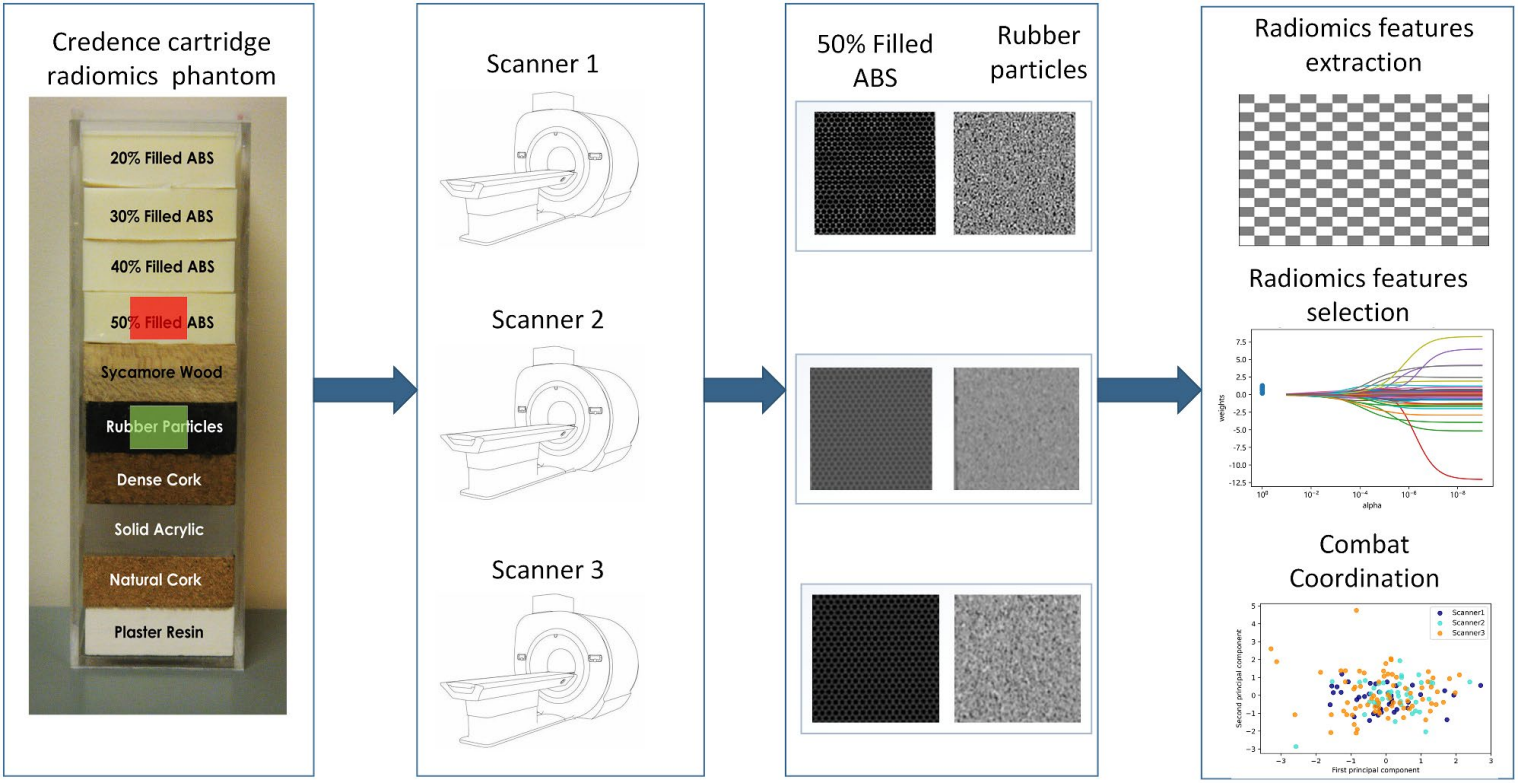
Flow chart of experimental design

As shown in Fig. 1, the segmentation of the region of interest was performed manually on the CCR phantom image. We use the open source software ITK-SNAP 3.6 to segment CT phantom images of different material cartridges [16]. Rubber particles were most commonly used in previous studies, and it was thought to be the closest to the texture of NSCLC [17, 18]. Therefore, in this study, 50% filled ABS and rubber particle cartridges were selected to extract texture features.

This study used cubes of the same size to avoid differences in radiomic features caused by differently shaped labeled regions. A total of 270 cubes of the same size were cut separately in a 50% filled ABS and rubber particles Cartridge. Areas with rubber pellets were labelled 0, and areas with 50% filled ABS were labelled 1. This study used 135 × 2 cubes scanned by GE, Philips, and Siemens as regions of interest (ROI) for radiomic feature extraction. In each ROI, all voxel values were linearly transformed to map between 0 and 1 by the Min-Max Normalization method. The image and segmentation data were stored in NIFTI format specially designed for neuroimaging with easy storage and readability.

### Radiomic feature extraction

The open-source library Pyradiomics 3.0.1 and Python 3.6.5 were employed to extract radiomic features [19], which were IBSI compliant [20]. The parameter for feature extraction was set as follows: the label value of the region of interest (ROI) in the label map was 0 or 1; the discretization value of the gray image level was 25; Voxel spacing was adjusted to 1 × 1 × 1 fixed size. This study excluded 2D shape features and 3D shape features because the ROI size in this study was the same. Table 3 shows that 22 Gy Level Co-occurrence Matrix (GLCM) features, 11 first-order statistical features, 16 Gy-Level Run Length Matrix (GLRLM), 14 Gy Level Size Zone Matrix (GLSZM), and 14 Gy Level Dependence Matrix (GLDM) features were extracted. All phantom image samples and radiomic features were divided into three groups according to the manufacturers, Siemens, Philips, and GE Healthcare.

**Table 3:** Name and classification of extracted radiomic features CCR Phantom

Category	Features
First orders	Percentile, Energy, Entropy, Interquartile Range, Kurtosis, Maximum, Mean Absolute Deviation, Mean, Median, Minimum, Range, Robust Mean Absolute Deviation, Root Mean Squared, Skewness, Total Energy, Uniformity, Variance,
Gray-Level Co-occurrence Matrix (GLCM)	Autocorrelation, Joint Average, Cluster Prominence, Cluster Shade, Cluster Tendency, Contrast, Correlation, Difference Average, Difference Entropy, Difference Variance, Joint Energy, Joint Entropy, Imc1, Imc2, Idm, Idmn, Id, Idn, Inverse Variance, Maximum Probability, Sum Entropy, Sum Squares
Gray-Level Run Length Matrix (GLRLM)	Gray Level non uniformity, Gray Level non uniformity Normalized, Gray Level Variance, High Gray Level Run Emphasis, Long Run Emphasis, Long Run High Gray Level Emphasis, Long Run Low Gray Level Emphasis, Low Gray Level Run Emphasis, Run Entropy, Run Length non uniformity, Run Length non uniformity Normalized, Run Percentage, Run Variance, Short Run Emphasis, Short Run High Gray Level Emphasis, Short Run Low Gray Level Emphasis
Gray Level Size Zone Matrix (GLSZM)	Gray Level non uniformity, Gray Level non uniformity Normalized, Gray Level Variance, High Gray Level Zone Emphasis, Large Area Emphasis, Large Area High Gray Level Emphasis, Large Area Low Gray Level Emphasis, Low Gray Level Zone Emphasis, Size Zone non uniformity, Size Zone non uniformity Normalized, Small Area Emphasis, Small Area, Small Area Low Gray Level Emphasis, Zone Entropy, Zone%, Zone Variance
Gray Level Dependence Matrix (GLDM)	Dependence Entropy, Dependence non uniformity, Dependence non uniformity Normalized, Dependence Variance, Gray Level non uniformity, Gray Level Variance, High Gray Level Emphasis, Large Dependence Emphasis, Large Dependence High Gray Level Emphasis, Large Dependence Low Gray Level Emphasis, Low Gray Level Emphasis, Small Dependence Emphasis, Small Dependence High Gray Level Emphasis, Small Dependence Low Gray Level Emphasis

### Combat compensation

The Combat algorithm was initially applied in genomics to adjust genetic data obtained from multiple batches of microarray experiments [10]. The Combat algorithm assumes that “batch effects” affect many genetic data in similar ways. Many studies state that “Batch effects” include experimental environment, work, technology, and operators. It is similar to the multicenter effect of radiomics features, which includes scanner manufacturer, scan parameters, and reconstruction algorithm. This study used a model-based location (mean) / scale (variance) adjustment method for multicenter radiomic feature adjustments. This method is generally modeled by normalizing the mean and variance. However, for the more complex case of radiomic features, a more general location/scale modeling framework is applied. The value of the radiomic feature g for the j sample at i scanner manufacturer can be written as:

1 $${\varvec{y}}_{\varvec{i}\varvec{j}\varvec{g}}= {\varvec{\alpha }}_{\varvec{g}}+ {\varvec{X}\varvec{\beta }}_{\varvec{g}}+{\varvec{\gamma }}_{\varvec{i}\varvec{g}} + {\varvec{\delta }}_{\begin{array}{c}ig \\ \end{array}}{\varvec{\epsilon }}_{\varvec{i}\varvec{j}\varvec{g}}$$

Where α is the average value of the feature ${\varvec{y}}_{\varvec{i}\varvec{j}\varvec{g}}$, $\varvec{X}$is the design matrix for the sample condition, ${\varvec{\beta }}_{\varvec{g}}$ is $\varvec{X}$ regression coefficient vector, ${\varvec{\gamma }}_{\varvec{i}\varvec{g}}$ is the additive form effect of different scanner manufacturers, $${\varvec{\delta }}_{\begin{array}{c}ig \\ \end{array}}$$ is the multiplier form effect of different scanner. The error term${\varvec{\epsilon }}_{\varvec{i}\varvec{j}\varvec{g}}$ follows a normal distribution with mean zero and variance ${\widehat{\sigma }}^{2}$.

2 $${\widehat{\varvec{y}}}_{\varvec{i}\varvec{j}\varvec{g}}= \frac{{\varvec{y}}_{\varvec{i}\varvec{j}\varvec{g}} -{\widehat{\varvec{\alpha }}}_{\varvec{g}}-{\varvec{X}\widehat{\varvec{\beta }}}_{\varvec{g}}-{\widehat{\varvec{\gamma }}}_{\varvec{i}\varvec{g}}}{{\widehat{\varvec{\delta }}}_{\varvec{i}\varvec{g}}} + {\widehat{\varvec{\alpha }}}_{\varvec{g}} +{\varvec{X}\widehat{\varvec{\beta }}}_{\varvec{g}}$$

The adjustment algorithm uses the least square method to estimate the model parameters,${\widehat{\varvec{\alpha }}}_{\varvec{g}}, {\widehat{\varvec{\delta }}}_{\varvec{i}\varvec{g}}, {\widehat{\varvec{\gamma }}}_{\varvec{i}\varvec{g}} \text{a}\text{n}\text{d} {\widehat{\varvec{\beta }}}_{\varvec{g}}$ can estimate the parameters $${\varvec{\alpha }}_{\varvec{g}},{\varvec{\beta }}_{\varvec{g}},{\varvec{\gamma }}_{\varvec{i}\varvec{g}},{\varvec{\delta }}_{\begin{array}{c}ig \\ \end{array}}$$ based on the model [10].

The mean of each radiomics feature corresponds to ${\widehat{\varvec{\alpha }}}_{\varvec{g}}$ in the formula. All radiomics features were divided into three batches corresponding to center i in the formula. The purpose was to investigate the performance of the Combat algorithm in removing noise from scanners manufactured by Siemens, Phillips, and GE Healthcare. The study compensated radiomic feature in each ROI using the python open-source library ComBatHarmonization https://github.com/Jfortin1/ComBatHarmonization.

### Machine learning model

Five machine-learning classification models were built to distinguish two different texture patterns. The selected radiomic features were divided into two groups: with the Combat group and without the Combat group, to investigate the impact of the Combat algorithm on subsequent modeling. Changes in the classification performance of five machine learning models between the two groups were investigated. It can assess the performance of subsequent analysis of radiomic features, after Combat compensation. For example, if the classification performance of the machine learning model degrades after the Combat algorithm adjusts the features. It indicates a negative impact of the Combat algorithm on the radiomic features. The dataset was randomly divided into 108 × 20 × 2 radiomic features as the training set and 27 × 20 × 2 as the test set (the format is the number of samples × number of features × two texture patterns, and the ratio is 8:2.

Figure 1 shows that all models are built using open-source scikit-learn 1.1.1. On the other hand, Table 4 shows that five different machine learning models were employed to evaluate the impact of the Combat compensation method on the classification performance of machine learning models. The five machine learning models include the least absolute shrinkage and selection operator (Lasso), logistic regression, random forests, support vector machines (SVM), and neural networks. The specific parameters of the model were set as follows.

**Table 4:** Parameter settings of five machine learning models

Model name	Model parameter	Parameter values
Least absolute shrinkage and selection operator (Lasso)	Lasso regression canonical term coefficient Maximum number of iterations	0.1 1000
Logistic regression	Logistic regression penalty term	‘l2’
Random forests	Number of random forest trees Maximum depth	100 8
Support vector machines (SVM)	SVM regularization parameter Kernel type Kernel coefficient	2 Radial basis function scale
Neural networks	Activation function Optimizer selection was ' Stochastic gradient descent’ Learning rate Maximum number of iterations Neural network composition	Rectified linear unit Stochastic gradient descent 0.01 200 1 input layer and5 hidden layers and 1 output layer

Many studies have demonstrated that the collinearity of radiomic features is an obstacle to improving the accuracy of model predictions. As a result, Lasso has been found to be the most efficient method to eliminate collinearity as it removes redundant features and filters the most relevant features for classification regardless of sample size [21]. Table 5 shows the 20 radiomic features most relevant to model classification predictions.

**Table 5:** Concordance test for ANOVA between different scanners

Features names	Without Combat	With Combat
Glcm Imc1	< 0.001	0.837
Glcm Inverse Variance	< 0.001	0.869
Gldm Low Gray Level Emphasis	< 0.001	0.975
Glszm Gray Level Variance	< 0.001	0.511
Glszm Zone%	< 0.001	0.978
Gldm Dependence non uniformity Normalized	< 0.001	0.894
First order Skewness	< 0.001	0.949
Glcm Sum Squares	< 0.001	0.889
Glcm Contrast	< 0.001	0.972
Glszm Large Area Low Gray Level Emphasis	< 0.001	0.878
First order Interquartile Range	< 0.001	0.975
Gldm Small Dependence Low Gray Level Emphasis	< 0.001	0.864
Gldm Large Dependence Low Gray Level Emphasis	< 0.001	0.910
First order Kurtosis	< 0.001	0.816
Glrlm Long Run Emphasis	< 0.001	0.876
Gldm Large Dependence High Gray Level Emphasis	< 0.001	0.939
First order Energy	< 0.001	0.838
Gldm Small Dependence High Gray Level Emphasis	< 0.001	0.907
Glszm Small Area Emphasis	< 0.001	0.842
Glcm Sum Entropy	< 0.001	0.905

### Statistical analysis

Statistical analysis was performed using the SPSS 25 software https://sourceforge.net/projects/spss/. The between-group variability results of the ANOVA test were used to characterize the effect of Combat on radiomics characteristics. In this study, a p-value of less than 0.05 was considered to have a significant difference, and a p-value of greater than 0.05 was considered to have no significant difference. The effect of Combat on the distribution of radiomic features was determined through principal component analysis. The study investigated changes in the distribution of radiomic features for each category before and after Combat. Distribution plots and boxplots of probability densities of radiomic features were used to present the results.

The model classification performance was evaluated by the area under the receiver curve (AUC), accuracy (ACC), precision, and recall. The feature importance ranking was investigated using logistic regression and random forest models. The five-fold cross-validation results of all classification models were recorded and statistically calculated using 95% confidence intervals.

## Result

### Radiomics feature compensation

ANOVA tests were performed across the radiomics features of the three manufacturers, and the results are shown in Table 5. The between-group difference was significant (*p* < 0.05) before Combat, indicating that the radiomics features were affected by scanners from different manufacturers. After Combat, the difference between groups was significant (p˃0.05), indicating that the Combat method successfully removed the influence of scanners from different manufacturers.

**Fig. 2:**
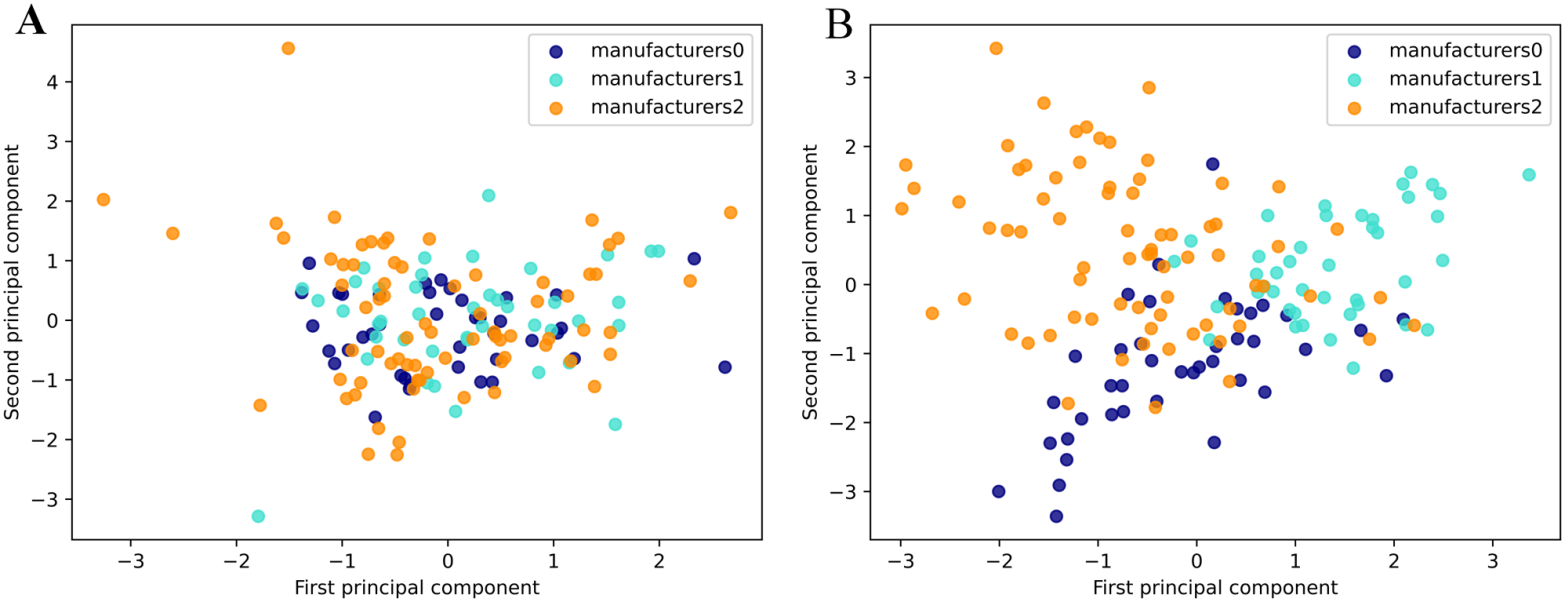
Principal component analysis of radiomic features, **A**: without Combat; **B**: with Combat

Figure 2 is the principal component analysis plot of radiomic features of the three groups of scanners before and after Combat. In Fig. 2A, the scanners are manufactured by Siemens, Philips, and GE, labeled as 0, 1, and 2 respectively. manufacturer 1 is distributed at the top, and manufacturer 2 and three are distributed at the lower left and lower right, respectively. These results show that the spatial distribution of the radiomic features of different manufacturers’ scanners is significantly different. In Fig. 2B, the radiomic features of the three manufacturers’ scanners are uniformly distributed, which shows that the combat method successfully reduced the variability from different manufacturers.

**Fig. 3:**
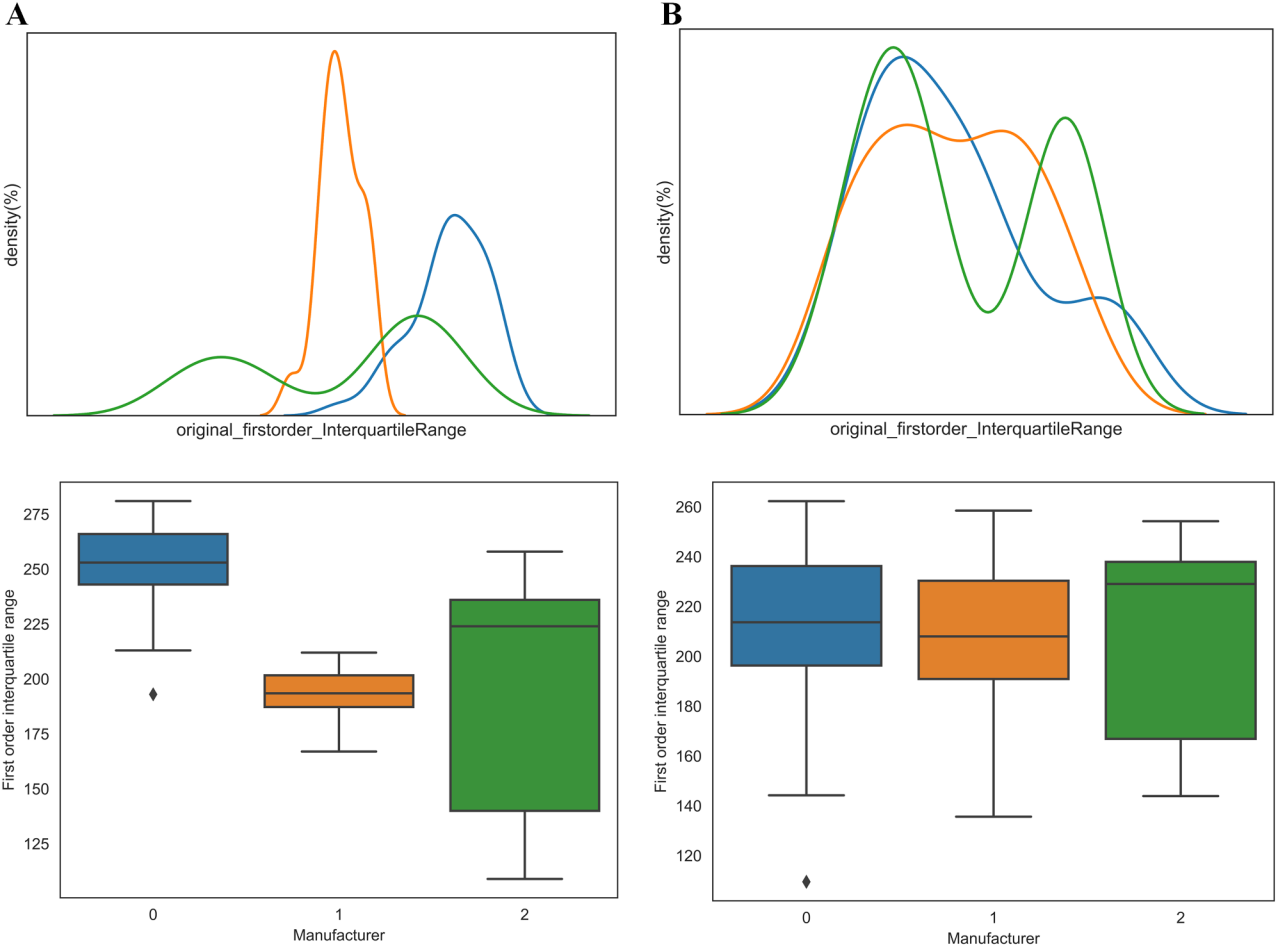
Density distribution of First Order interquartile range in with and without Combat. **A**: without Combat; **B**: with Combat

Figure 3 shows the probability density functions and boxplots of the texture features. The first order features were shown here (GLDM, GLCM, and GLRLM features were shown in Supplementary Figs. 1, 2, and 3). The three colors represent the radiomics features of the three manufacturer’s scanner groupings. Figure 3A shows that the distribution of radiomics features varied significantly among the three groups. Boxplots also reveal notable differences between groups, which can impact subsequent statistical analyses and model accuracy. Figure 3B shows the distribution after Combat compensation. Combat compensation removes differences in the distribution of radiomics features between scanners from different manufacturers. The shapes of the distributions of the same set of features after Combat are roughly the same. It partly demonstrates that Combat maintains classification specificity while removing unwanted noise from different manufacturers.

The results indicate that radiomic features are sensitive to different CT scanners and manufacturers, leading to poor stability and robustness of these features. The Combat algorithm successfully removes the variability in radiomic features caused by different scanner manufacturers. It suggests that the Combat algorithm can harmonize the distribution of radiomic features and eliminate the multicenter effects of radiomic features.

### Machine learning models

**Fig. 4:**
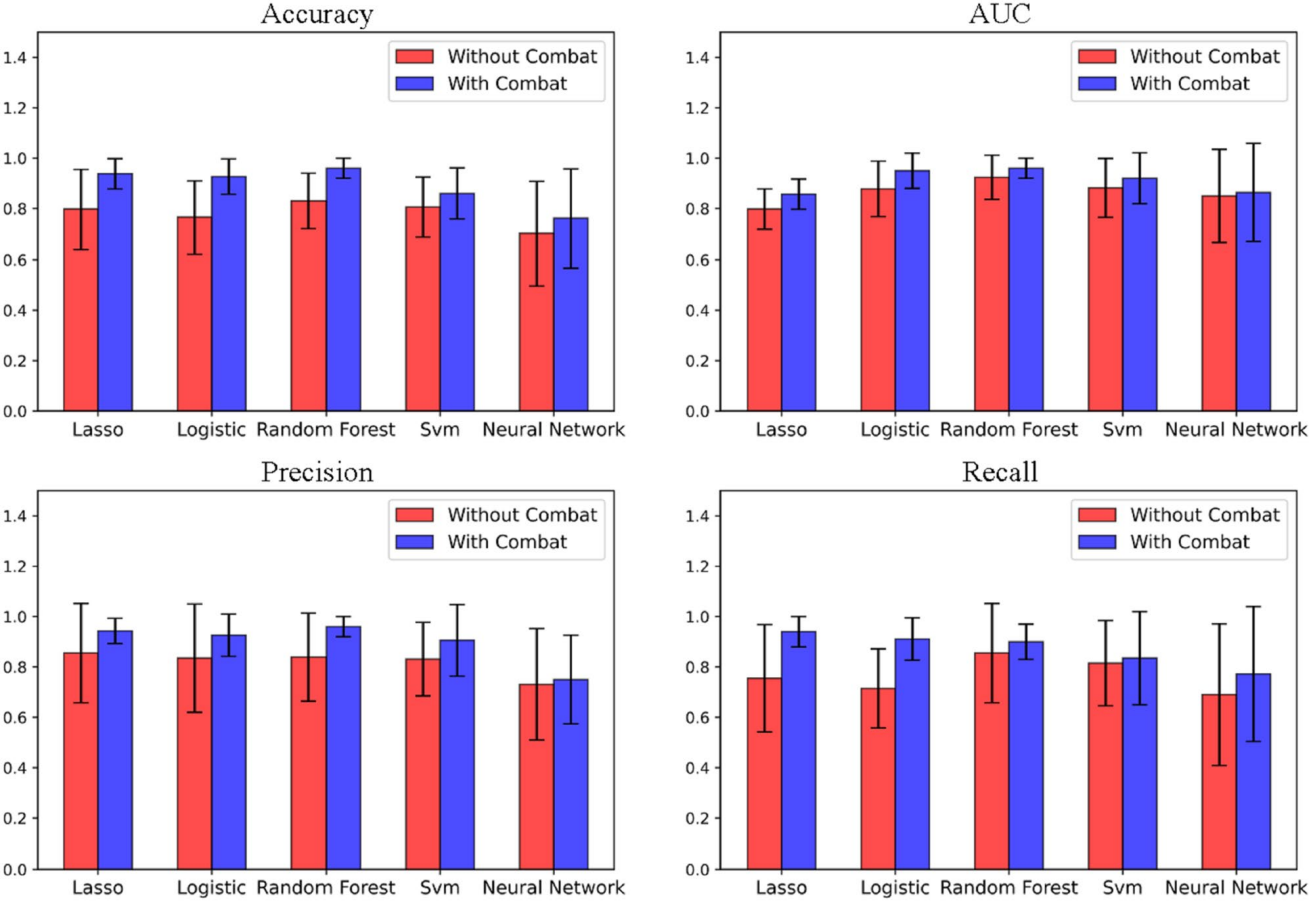
Performance metrics evaluation of different model performance indicators in with and without Combat

Figure 4 shows the classification performance of five machine learning models (Lasso, Logistic, Random Forest, SVM, and Neural network) for radiomics features in two different regions. The red bars show the results of machine learning classification of radiomics features before Combat compensation, whereas the blue bars represent the results of machine learning classification of radiomics features after Combat compensation. The error bars represent the range of validation errors. Compensation of the radiomic features using Combat improved the classification performance of five machine learning models.

The logistic regression and the random forest models outperformed the other three models. Maybe due to the small dataset size and overfitting, the neural network’s classification performance was lower than that of all other models. The impact of radiomic features on the classification performance of machine learning models before and after Combat was compared. The study found that the accuracy and precision of the model were significantly improved. After Combat, the error margins of most of the model classification results were reduced, demonstrating that the Combat method can enhance the accuracy and stability of model classification.

**Fig. 5:**
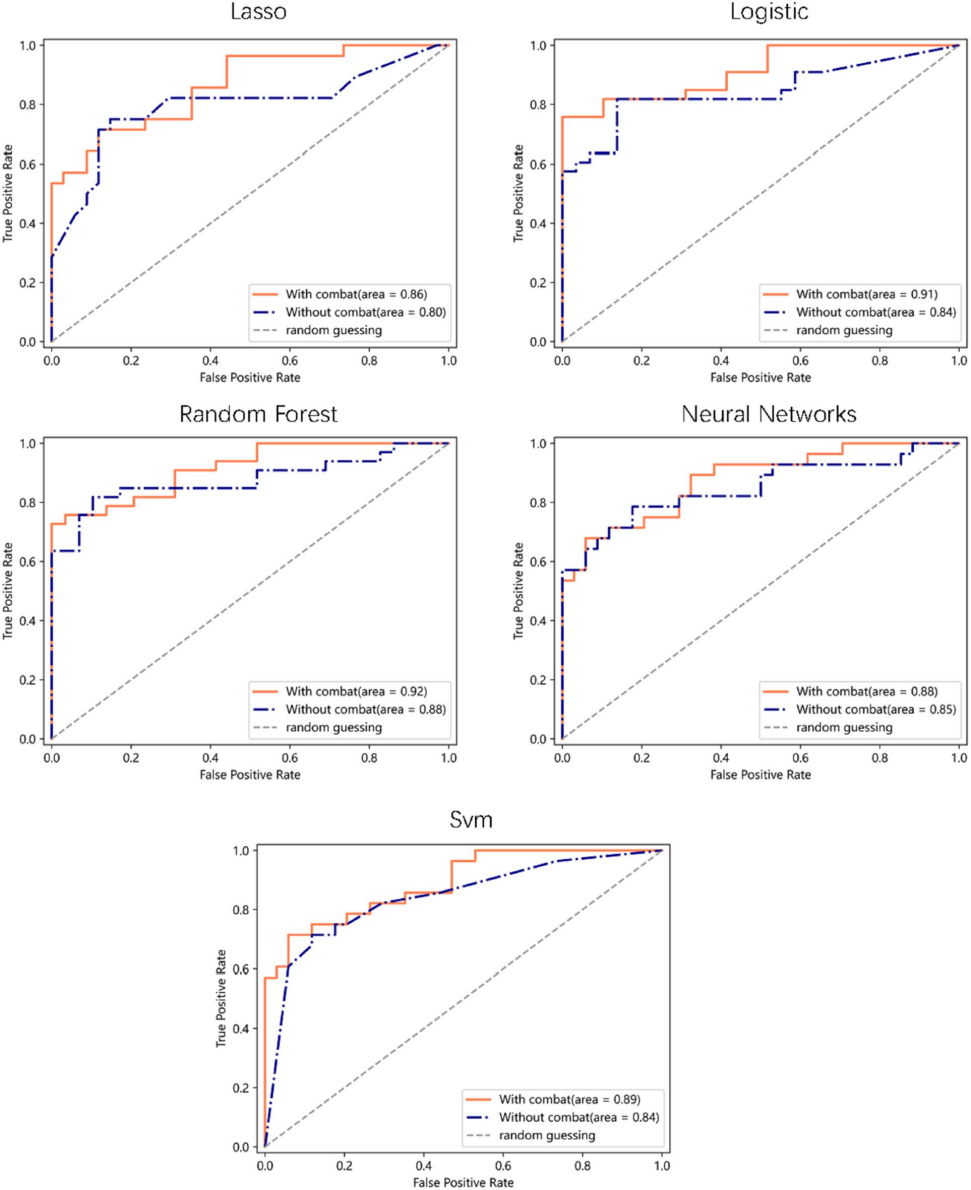
ROC curves of five machine learning models in with and without Combat

Figure 5 shows the optimal ROC curves of five machine learning models for classification tasks, where the blue curve is before Combat, and the red curve is after Combat. The ROC values of logistic regression and random forest models were 0.84 and 0.88 before Combat 0.91 and 0.92 after Combat.

Different radiomic features contribute differently to machine learning model classification. After Combat compensation, the importance of the contribution of radiomic features changes, and the magnitude of the change can reveal the impact of Combat on radiomic features. In Supplementary Figs. 4 and 5, changes in the importance ranking of radiomic features were investigated, after Combat compensation,

The importance of the variable was derived from the value of the coefficient of the variable in the logistic regression analysis. Supplementary Fig. 4A features importance statistics before Combat, and Supplementary Fig. 4B shows feature importance statistics after Combat. The results show that texture features such as GLCM are more important than statistical features. The study also found that the importance rankings for most features changed slightly, indicating that the Combat method did not significantly affect the specificity of radiomic features. The results also show that the proportion of feature importance in the classification contribution is unchanged. However, the study observed fluctuations in the importance ranking of a few radiomic features, such as the first-order interquartile range, which shows that the Combat algorithm still requires further refinement to adapt to the multi-center effect compensation problem of radiomic features. Supplementary Fig. 5 shows that radiomic feature importance changed after using Combat. The results of the random forest model are consistent with those of logistic regression.

In this phantom CT dataset, there was a modest improvement in the AUC value of the harmonized features. It suggests that the Combat algorithm may potentially enhance the classification performance of radiomic machine learning models.

### Discussion

Radiomics features are sensitive to medical image data from different centers, which vary with the acquisition equipment, manufacturers, acquisition parameters, and reconstruction kernels [22]. Several radiomics studies have analyzed medical images from several medical institutions and different scanner models. It has been found that the multicenter problem is a major challenge interfering with the application of radiomic features in large-scale multicenter data and clinical practice.

This study investigated the impact of the Combat algorithm in removing the variability of CT phantom data from different manufacturers. The study used the PCA and ANOVA to examine the influence of different manufacturers on radiomic features. It was observed that the sample distribution of the principal component analysis was different for different manufacturers, which indicated that different scanner manufacturers resulted in variations among radiomic features. Radiomic features were also sensitive to scanner [23, 24], reconstruction kernel [25, 26], and scan parameters [27].

PCA revealed that the distribution differences between groups of radiomics feature disappeared after the Combat algorithm adjusted the radiomics features. The ANOVA consistency test showed that the differences in radiomics features between different groups disappeared, and the p-values of all features changed from less than 0.05 to greater than 0.05. Johnson believed that the Combat algorithm removes the variability of different batches and preserves its biological specificity [10]. Many studies have also demonstrated that the Combat algorithm has a good adjustment effect for radiomic features from different voxel sizes, reconstruction kernels, and scanning protocols [11, 28].

Microarray data for genes are often influenced by in the types of chips, samples, and labels [10]. Similarly, radiomics feature data often vary between scanners, scanner manufacturers, and other parameters. The Combat algorithm assumes that the distribution of radiomics features generally follows a location (mean)/size (variance) distribution. Combat uses modeling to fit the distributions and errors of radiomics features and then estimates the model parameters and errors. The radiomics features of scanners from different manufacturers were defined as different batches and adjusted according to Eq. 2.

Table 1 shows the 50% ABS resin and rubber particle cartridge areas in the cartridge CT phantom data marked as ROIs. 100 radiomic features were extracted from the ROI region. There are some features in the radiomics that are redundant, and cross-correlated features need to be excluded and it will also bias the subsequent analysis [29]. Lasso regression was utilized to select the radiomic features most relevant to model predictions. Many studies show that the Lasso regression model is the most efficient variable selection method [30].

Five frequently used machine learning models Lasso, logistic regression, random forest, SVM, and neural network, were designed to distinguish radiomics features. The performance of these five machine learning models was compared before and after Combat. The results show that Combat can not only remove unwanted variation from scanners but also can improve model classification accuracy.

As shown in Figs. 3, 4 and 5, the Combat algorithm aligns the centers and scales of the radiomic features’ distributions by standardizing the feature distributions. This helps to remove the variability in radiomic features. The evaluation of radiomic machine learning model classification performance results demonstrates that the Combat algorithm may improve the classification performance of machine learning models. One possible reason is that the Combat algorithm mitigates the interference of unfavorable factors, such as scanner models, on radiomic features. However, this result is currently only tested on this whole-body dataset, and rigorous conclusions require comprehensive validation and assessment.

Fanny Orlhac and his colleagues found that the relative positions and shapes of the density distributions of different groups of features were the same before and after Combat [11]. They believe this indicates that the properties of the radiomic signature have not changed after Combat compensation. On the other hand, Jean-Philippe and his colleagues applied Combat to compensate cortical thickness measurements from different scanners [13]. Demonstrated that the Combat algorithm successfully removed noise from cortical thickness measurements from different scanners. In addition, they verified that the correlation of cortical layer thickness with age persisted after Combat compensation. This study investigated changes in feature importance before and after Combat based on logistic regression and random forests. We found that the importance of texture features was altered as a result of Combat’s adjustments. Texture features were found to be influenced by different scanners [6]. Although the model evaluation method achieved good performance, the Combat method need to be improved to ensure stability of features.

This study also has some shortcomings. Combat algorithm can only adjust the existing data, but cannot be applied to adjust new data. Ronrick and his colleagues tried to use deep learning to fit Combat’s process so that it could be applied to new data [31]. It will be interesting attempt, but improving the Combat algorithm is more direct and efficient. In other words, if the compensation performance of the Combat algorithm is not improved, there is no prospect of using another model to simulate this process. The dataset used in this study were limited to phantom CT. We hope that in future studies, improvements to the structure of the Combat algorithm can be made. The aim is to develop a feature variability harmonization algorithm that is specifically applicable to the field of radiomics.

### Conclusion

This study collected CT phantom images from different scanners manufactured by different companies. In total, 100 radiomic features were extracted. The ANOVA test and feature probability density distribution results show that the Combat algorithm successfully removes the noise of radiomics features from the different scanners. the Combat algorithm improved the performance of subsequent modeling analysis of radiomic features. However, whether the Combat algorithm can improve the robustness and classification performance of radiomic machine learning models in clinical disease CT images still requires further validation.

### Electronic supplementary material

Below is the link to the electronic supplementary material.

Supplementary Material 1

## Declarations

### Data availability

The data for this study were collected from public datasets. https://wiki.cancerimagingarchive.net/pages/viewpage.action? pageId=39,879,218.

## Abbreviations

ABS: Acrylonitrile Butadiene Styrene
ANOVA: Analysis of Variance
AUC: Area Under THE Receiver Curve
CCR: The Credence Cartridge Radiomic
CT: Computed Tomography
FOV: Field of View
GLCM: Gray Level Co-occurrence Matrix
GLRLM: Gray-Level Run Length Matrix
GLSZM: Gray Level Size Zone Matrix
GLDM: Gray Level Dependence Matrix
Lasso: The Least Absolute Shrinkage and Selection Operator
NIFTI: Neuroimaging Informatics Technology Initiative
NSCLC: Non-Small Cell Lung Cancer
ROI: Regions of Interest
ROC: Receiver Operating Characteristic Curve
SVM: Support Vector Machines
