# Supplementary material for: The impact of the combat method on radiomics feature compensation and analysis of scanners from different manufacturers
Source: BMC Med Imaging. 2024 Jun 6;24:137. doi: 10.1186/s12880-024-01306-4 (PMC11157873; doi:10.1186/s12880-024-01306-4)
Supplement: Supplementary file 1 — Supplementary Material 1 [file 12880_2024_1306_MOESM1_ESM.docx]

**Supplementary material**


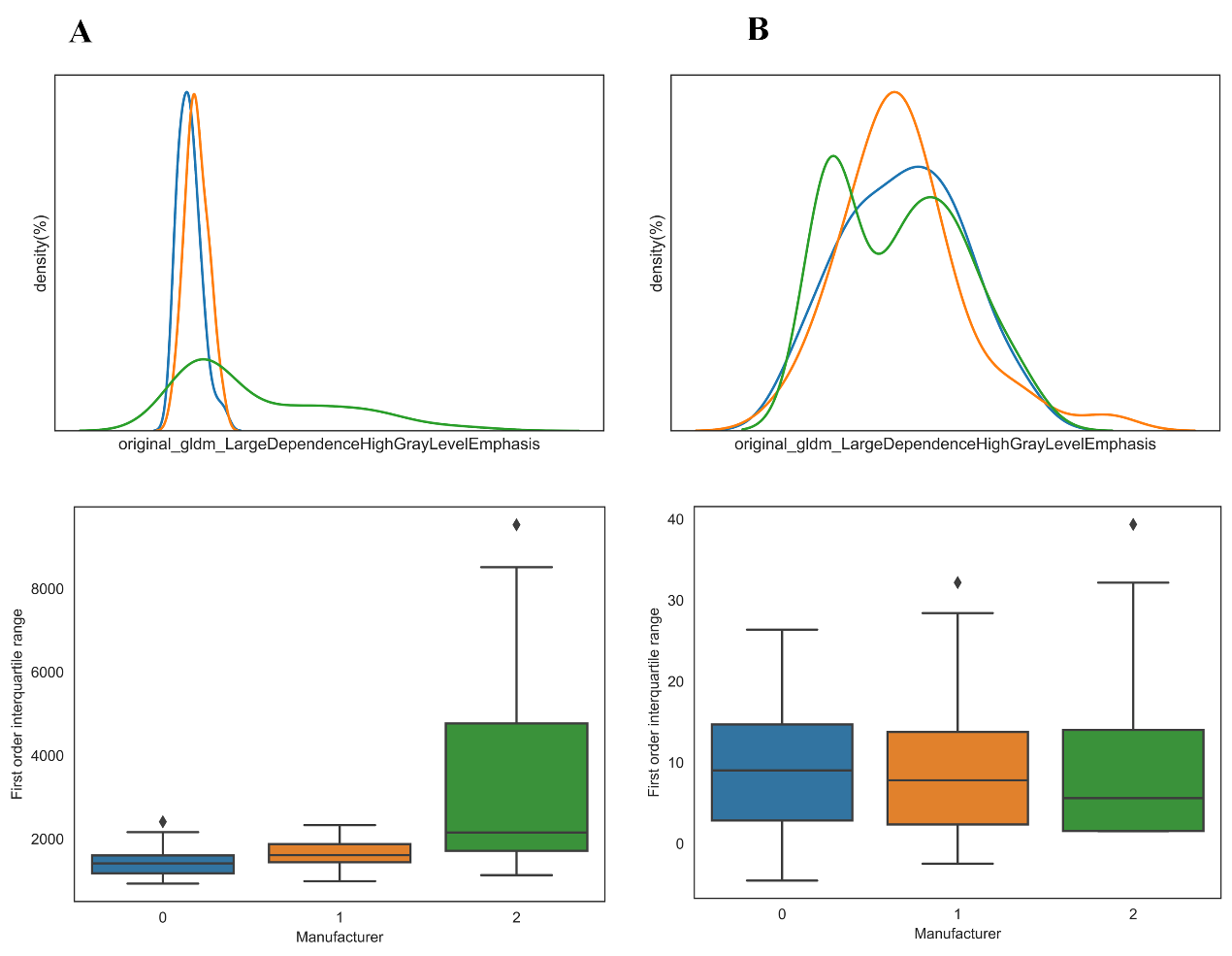


Supplementary Figure 1. Density distribution of GLDM in with and without Combat. A: without Combat; B: Combat by Scanners from different manufacturers.


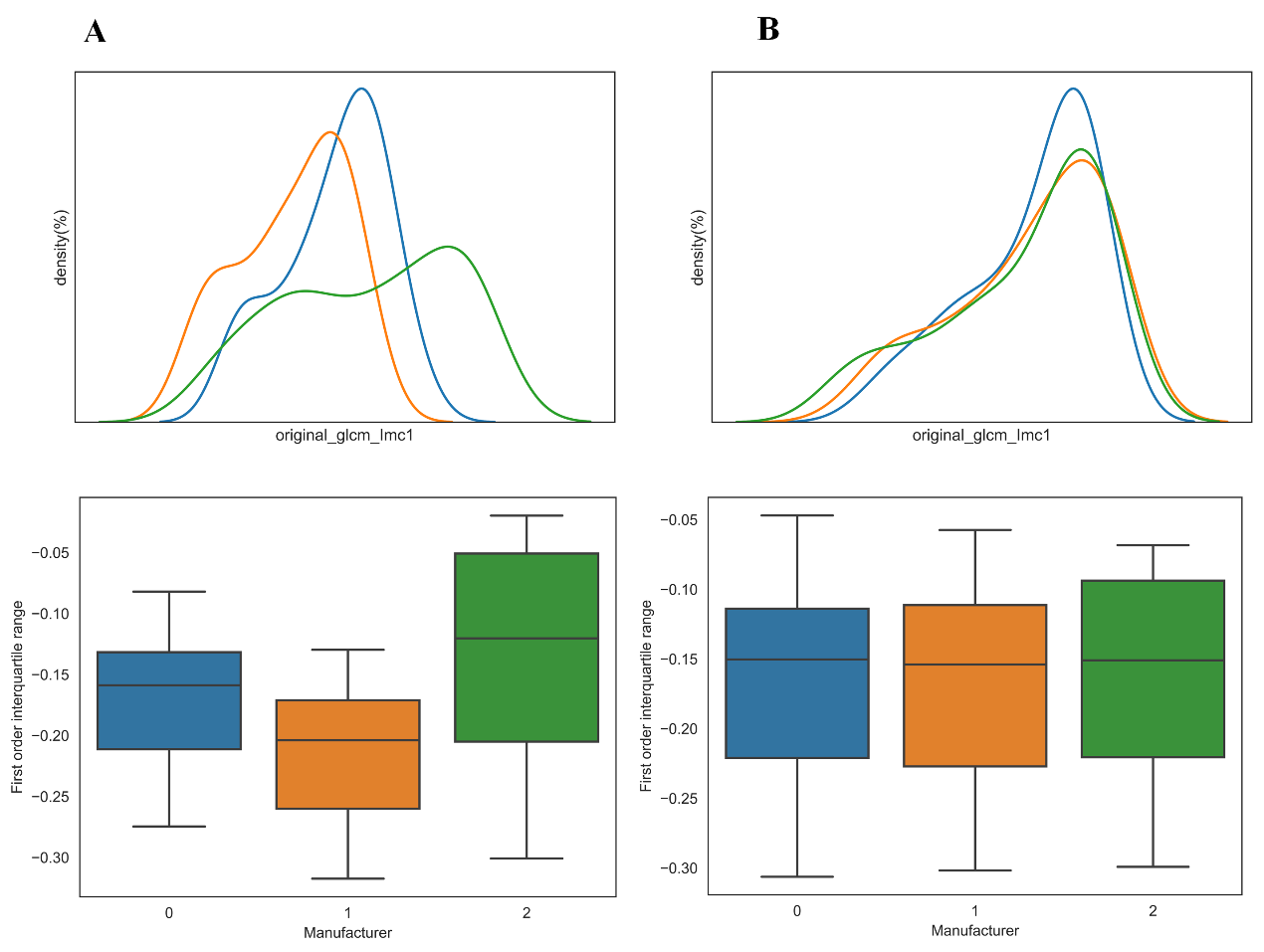


Supplementary Figure 2. Density distribution of GLCM in with and without Combat. A: without Combat; B: Combat by Scanners from different manufacturers.


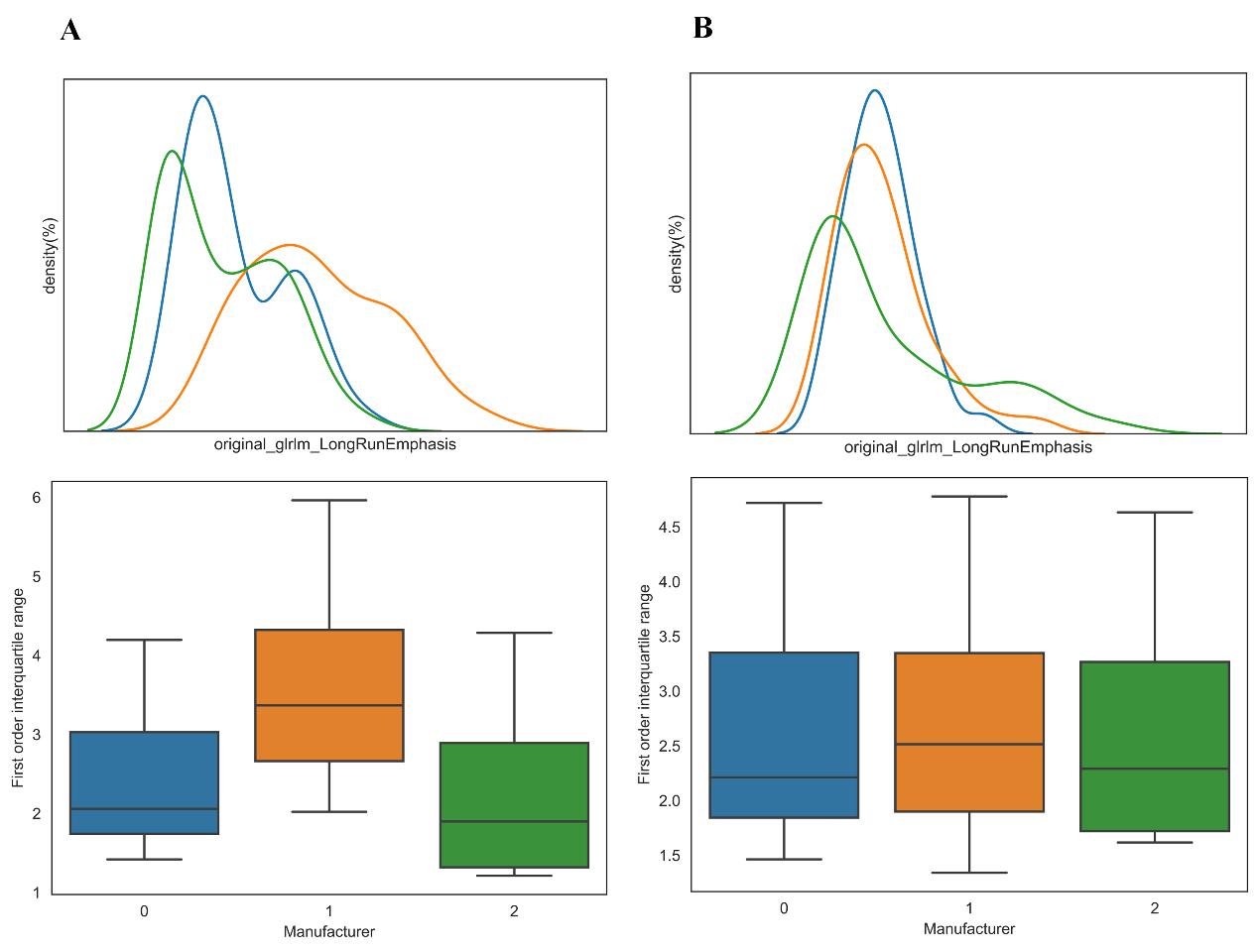


Supplementary Figure 3. Density distribution of GLRLM in with and without Combat. A: without Combat; B: Combat by Scanners from different manufacturers.


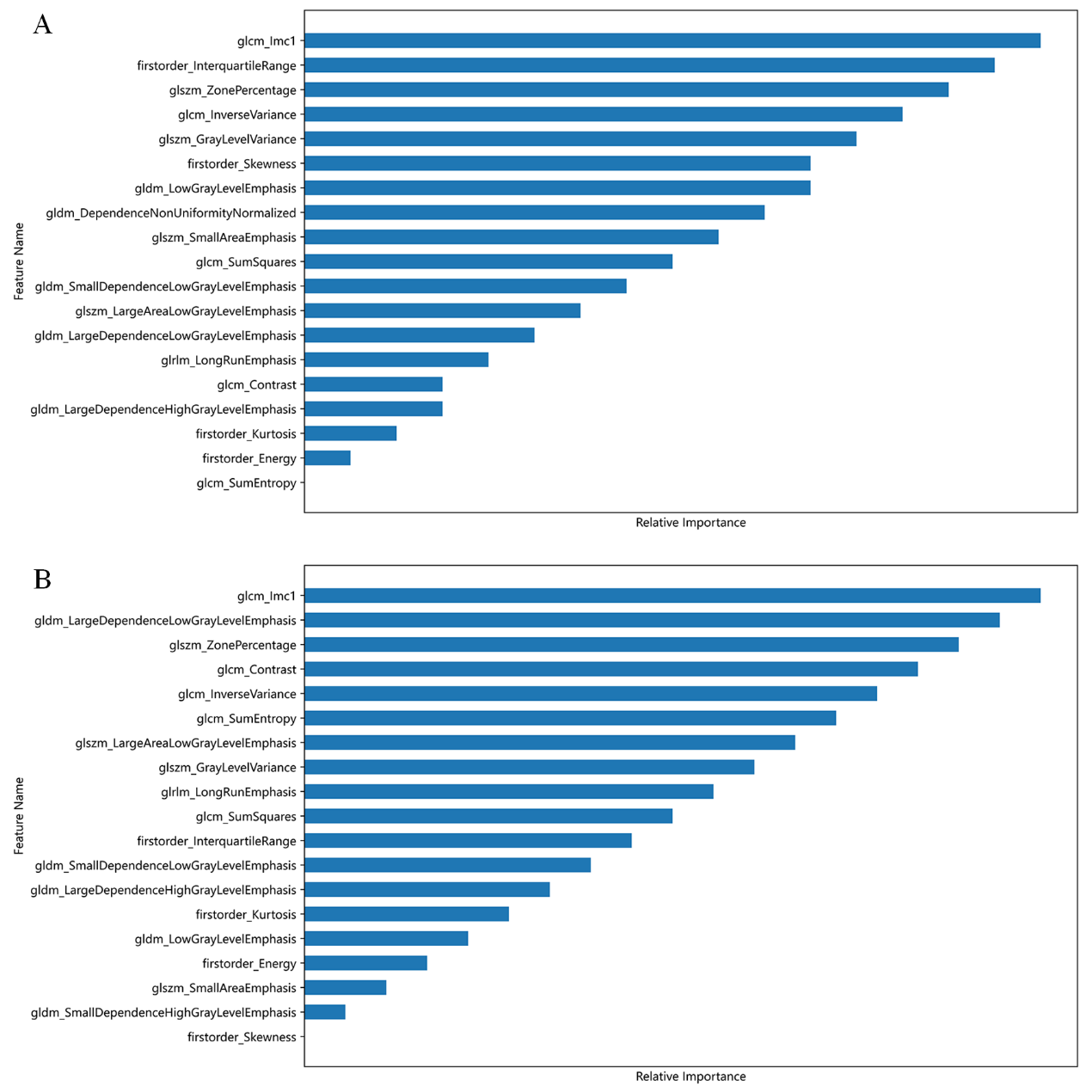


Supplementary Figure 4. Logistic regression model radiomic feature importance ranking in with and without Combat, A: without Combat; B: with Combat


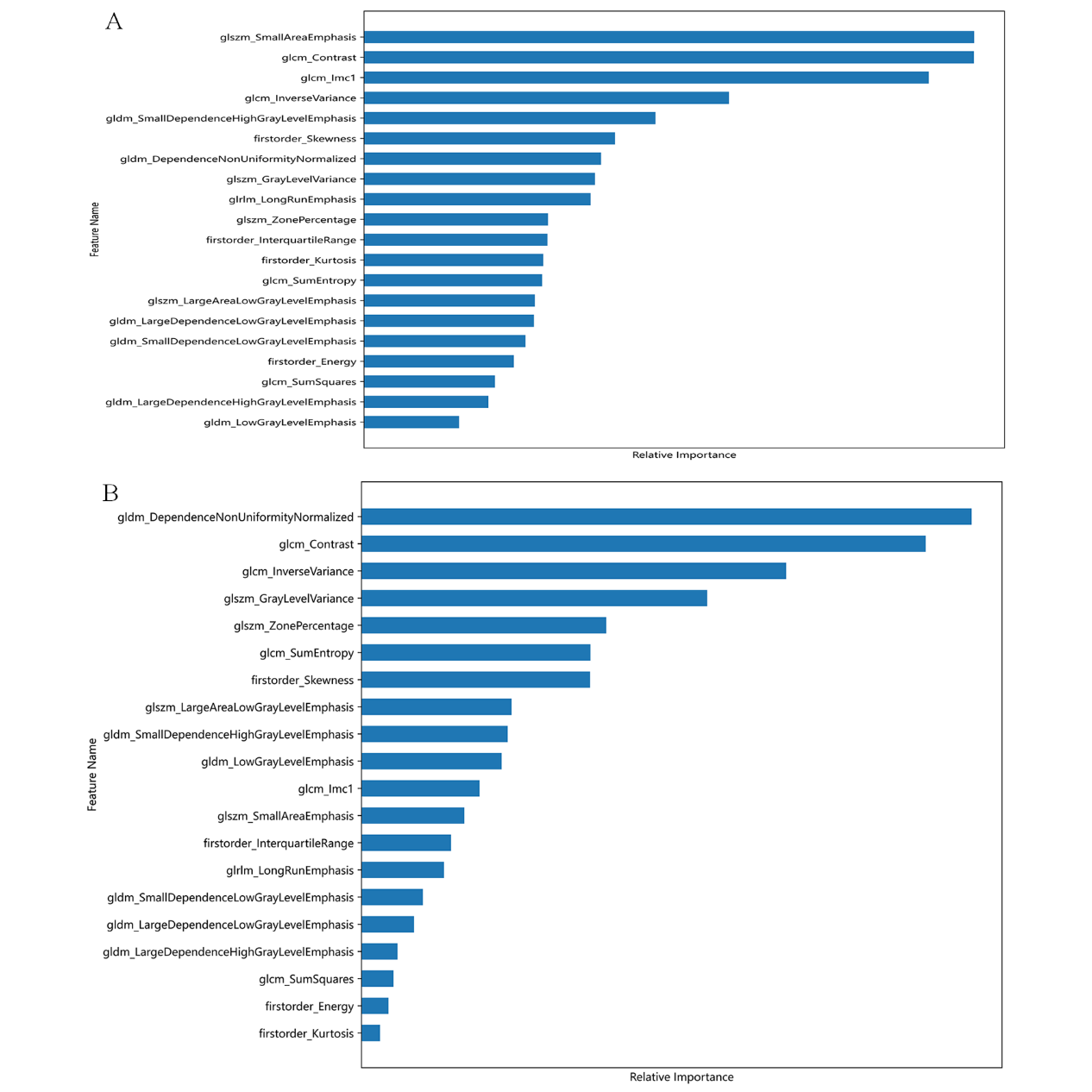


Supplementary Figure 5. Random Forest model radiomic feature importance ranking in with and without Combat, A: without Combat; B: with Combat
